# Supplementary material for: Reversible First-Order Single Crystal to Single Crystal Thermal Phase Transition in [(CH3)3CNH3]4[V4O12]
Source: Materials (Basel). 2022 Aug 17;15(16):5663. doi: 10.3390/ma15165663 (PMC9416169; doi:10.3390/ma15165663)
Supplement: Supplementary file 1 [file materials-15-05663-s001.zip › materials-1850155-supplementary.pdf]

Article

Reversible First-Order Single Crystal to Single Crystal Thermal Phase Transition in $[(CH_3)_3CNH_3]_4[V_4O_{12}]$

Pablo Vitoria [1], Ana San José Wéry [2], Leire San Felices [3], Laura Bravo-Garcia [1], Estibaliz Ruiz-Bilbao[1] José Manuel Laza [4], José Luis Vilas [4, 5] and Juan M. Gutiérrez-Zorrilla [1, 5, *]

1 Departamento de Química Orgánica e Inorgánica, Facultad de Ciencia y Tecnología, Universidad del País Vasco UPV/EHU, P.O. Box 644, 48080 Bilbao, Spain
2 Facultad de Ciencias y Artes, Universidad Católica de Ávila, c/Canteros s/n, 05005 Ávila, Spain
3 Servicios Generales de Investigación SGIker, Facultad de Ciencia y Tecnología, Universidad del País Vasco UPV/EHU, P.O. Box 644, 48080 Bilbao, Spain
4 Departamento de Química Física, Facultad de Ciencia y Tecnología, Universidad del País Vasco UPV/EHU, P.O. Box 644, 48080 Bilbao, Spain
5 BCMaterials, Parque Tecnológico de Bizkaia, Edificio 500, 48160 Derio, Spain.
* Correspondence: juanma.zorrilla@ehu.eus.; Tel.: +34 946015522

Synthesis and characterization of $[(CH3)_3CH_3]_4[V_4O_{12}]$. To a stirred aqueous suspension (25 mL) of V_2O_5 (5.82 g; 32 mmol), an excess of tert -butylamine (10.0 mL; 94 mmol) was added dropwise. The mixture was stirred at room temperature for 15 min. Then, the solution was filtered to remove insoluble black residues, and the resulting colorless solution was heated under reflux for 2 h. After 4 days, very nice prismatic colorless crystals of $[(CH_3)_3CNH_3]_4[V_4O_{12}]$ were obtained. Anal. Calcd for $C_{16}H_{48}N_4O_{12}V_4$: C 27.76; H 6.99; N 8.09; $V(V_2O_5)$ 52.54. Found: C 27.81; H 6.99; N 8.02; V(V 2 O 5) 51.80. D 0) 1.39(1). IR (cm^{-1}); Figure S1: 890 (s, br) [ν_s ($V(O_t)_2$)]; 810 (ν_s, br) [ν_{as} ($V(O_t)_2$)]; 500(w) [ν_{as} (V-O_b-V)]; 460 (m) [ν_s (V-O_b-V)]; 375 (w) [$\delta(VO_2)$].

Table S1. Selected bond lengths (Å) of the $[V_4O_{12}]^{4-}$ anion in compound **1** for the measures at **HTP** and **LTP**.

	1c_173	1c-163	1c-153	1c_143	1c_133	1h_143	1h_153	1h_163	1h_173	1h_183
V1–O1	1.6351(18)	1.6331(18)	1.641(2)					1.6337(18)	1.6365(17)	1.6319(18)
V1–O2	1.775(2)	1.759(2)	1.756(3)					1.759(2)	1.777(2)	1.770(2)
V1–O2^{i}	1.762(2)	1.776(2)	1.775(3)					1.775(2)	1.762(2)	1.760(2)
V1–O1A				1.613(2)	1.620(2)	1.617(2)	1.613(2)			
V1–O1B				1.647(2)	1.6537(19)	1.652(2)	1.645(2)			
V1–O12				1.781(2)	1.786(2)	1.783(2)	1.778(2)			
V1–O14				1.787(2)	1.789(2)	1.786(2)	1.784(2)			
V2–O2A				1.617(2)	1.622(2)	1.618(2)	1.616(2)			
V2–O2B				1.650(2)	1.654(2)	1.651(2)	1.645(2)			
V2–O12				1.787(2)	1.7895(19)	1.784(2)	1.784(2)			
V2–O23				1.779(2)	1.782(2)	1.782(2)	1.778(2)			
V3–O3A				1.6529(19)	1.6567(18)	1.651(2)	1.651(2)			
V3–O3B				1.652(2)	1.633(2)	1.631(2)	1.633(2)			
V3–O23				1.775(2)	1.785(2)	1.778(2)	1.775(2)			
V3–O34				1.771(2)	1.7723(19)	1.770(2)	1.768(2)			
V4–O4A				1.643(2)	1.6513(19)	1.656(2)	1.645(2)			
V4–O4B				1.631(2)	1.632(2)	1.629(2)	1.629(2)			
V4–O34				1.782(2)	1.7873(19)	1.787(2)	1.78742)			

V4–O14				1.763(2)	1.770(2)	1.768(2)	1.765(2)			

Symmetry codes. ^{i}: –y, *x*, *z*.

Table S2. Selected hydrogen bond lengths (D – H, H...A and D...A (Å)) and angles (D - H...A (°)) in compound **1-HTP** (at 1h_173) and **1-LTP** (at 1c_143).

	Donor-H....Acceptor	D - H	H...A	D...A	D - H...A
**1-LTP**	N1--H1A O4^{i}	0.92	1.86	2.770(3)	171
	N1--H1B O4^{ii}	0.85	2.33	3.076(3)	145
	N1--H1C O4^{iii}	0.85	2.33	3.076(3)	145
**1-HTP**	N1A--H1AA O4A^{iv}	0.89	1.89	2.782(3)	175
	N1A--H1AB O1B^{v}	0.89	1.92	2.786(3)	164
	N1A--H1AC O1B^{ii}	0.89	2.00	2.840(3)	158
	N1B--H1BA O3A^{vi}	0.89	1.91	2.792(3)	174
	N1B--H1BB O4B^{vii}	0.89	1.86	2.738(3)	170
	N1B--H1BC O2B^{ii}	0.89	2.02	2.851(3)	154
	N1C--H1CA O2A^{viii}	0.89	1.88	2.762(3)	175
	N1C--H1CB O3B^{ix}	0.89	1.90	2.783(3)	172
	N1C--H1CC O3A^{ii}	0.89	2.01	2.862(3)	159
	N1--H1CC O3B^{ii}	0.89	2.55	3.213(3)	132'
	N1D--H1DA O1A^{x}	0.89	1.87	2.747(3)	170
	N1D--H1DB O2B^{xi}	0.89	1.88	2.772(3)	176
	N1D--H1DC..O4A^{ii}	0.89	2.14	2.958(3)	152
	N1D--H1DC..O4B^{ii}	0.89	2.44	3.160(3)	138'

Symmetry codes ^{i}= ½-x, 1/2-y, 1/2+z, ^{ii}= x, y , z; ^{iii}= x, y ,-z; ^{iv}=3/4-y,3/4+x,3/4-z; ^{v}= 1/2-x,3/2-y,1/2-z; ^{vi}= 1/4+y,3/4-x,3/4-z; ^{vii}=1/2+x,y,1/2-z; ^{viii}=1/2-x,1/2-y,1/2-z; ^{ix}=3/4-y,-1/4+x,3/4-z; ^{x}= -3/4+y,3/4-x,3/4-z; ^{xi}=-1/2+x,y,1/2-z.

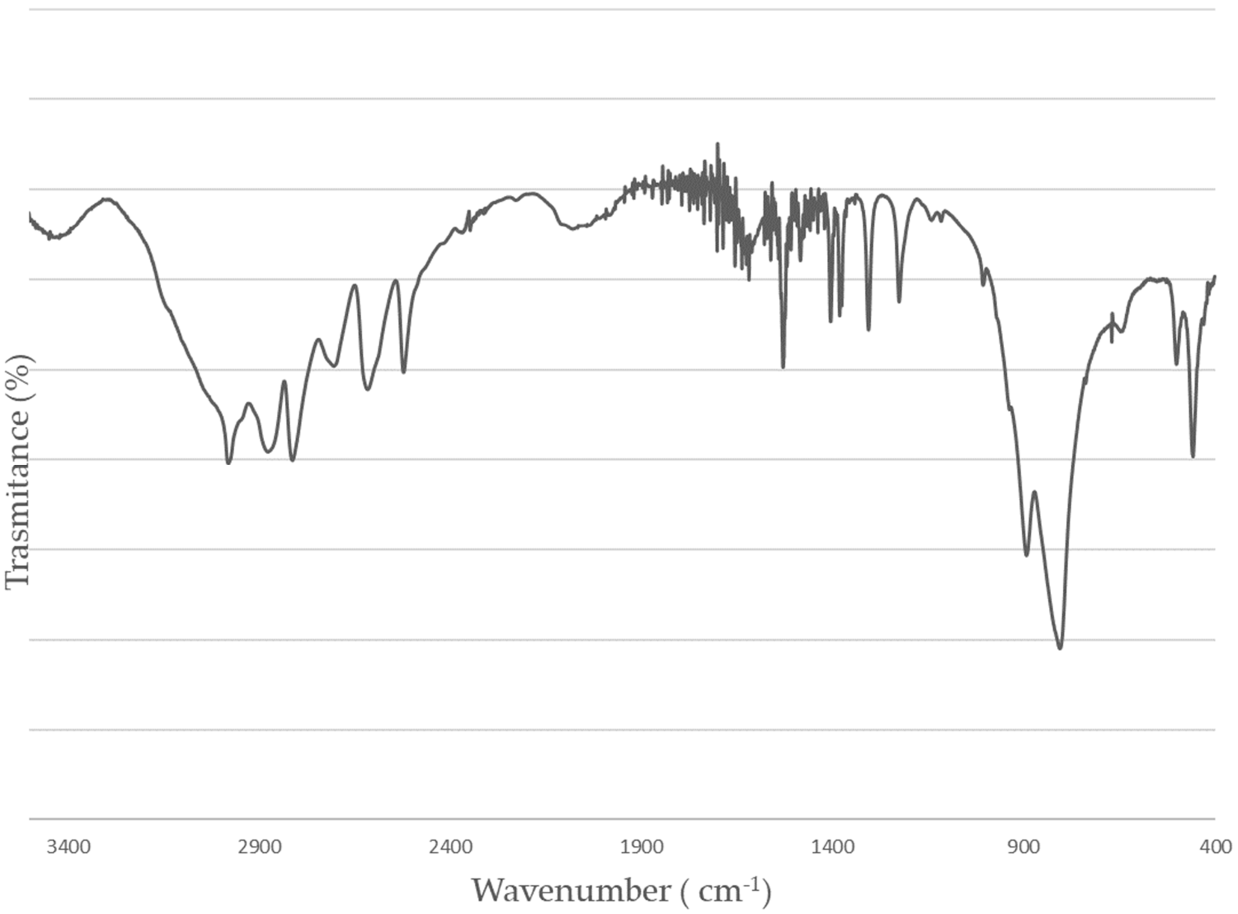

Figure **S1.** FTIR spectra from $[(CH3)_3CH_3]_4[V_4O_{12}]$ (**1h-RT**).

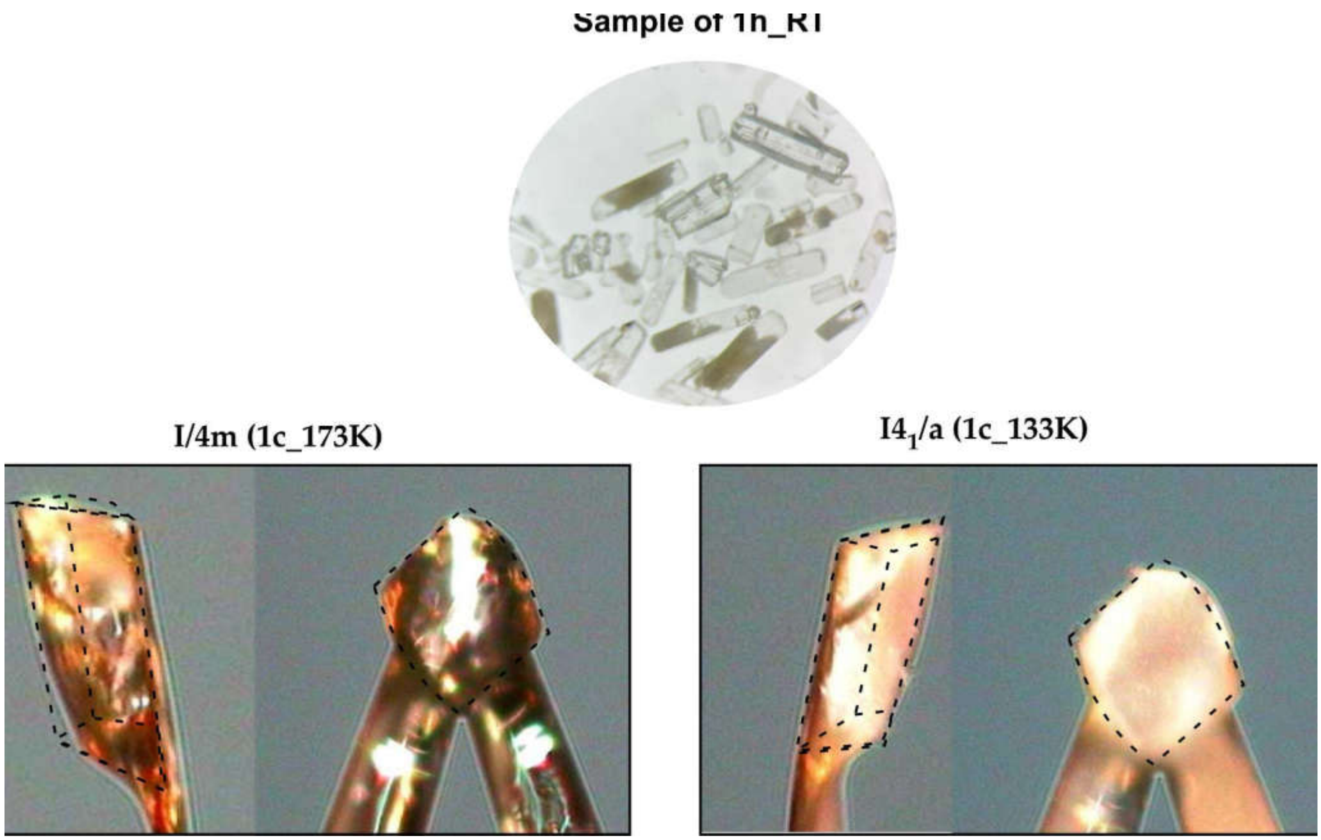

Figure S2. Image of the crystal sample and a single crystal of it placed on the diffractometer pen.

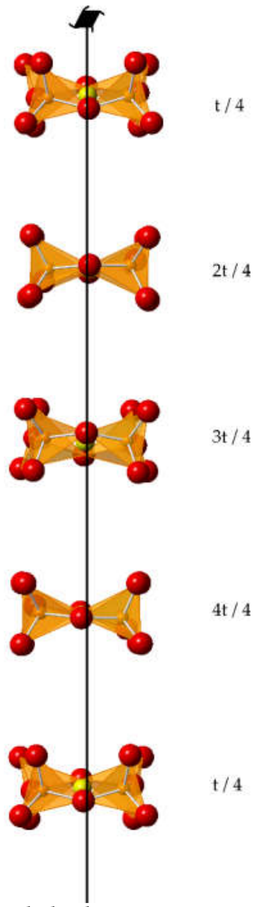

Figure S3. Graphical representation of the rotation in a helical quaternary axis.

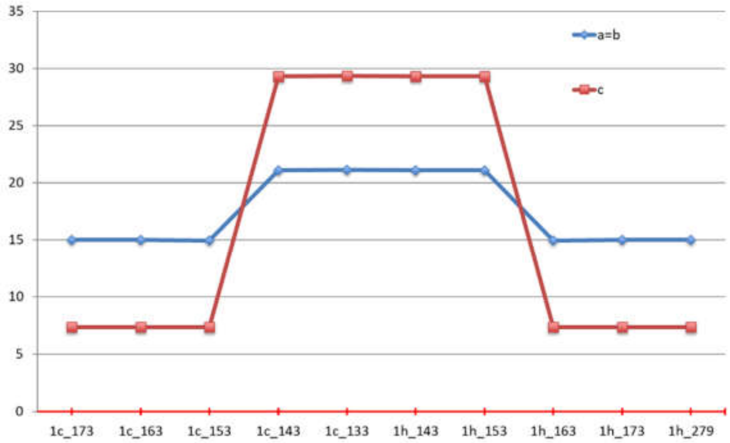

Figure S4. Graphical representation of the dependence of cell parameters (a=b in blue and c in red) with temperature.
